# A Causal Network Analysis of the Fatty Acid Metabolome in African-Americans Reveals a Critical Role for Palmitoleate and Margarate

**DOI:** 10.1089/omi.2016.0071

**Published:** 2016-08-01

**Authors:** Azam Yazdani, Akram Yazdani, Eric Boerwinkle

**Affiliations:** Human Genetics Center, University of Texas Health Science Center at Houston, Houston, Texas.

## Abstract

Fatty acids are important sources of energy and possible predictors and etiologic factors in many common complex pathologies such as cardiovascular disease, diabetes, and certain forms of cancers. While fatty acids are thought to covary with each other, their underlying causal networks have not been fully elucidated. This study reports the identification and analysis of a statistical causal network among 15 mostly long-chain fatty acids. In an African-American population sample and using the Genome granularity-Directed Acyclic Graph (GDAG) algorithm, we determined directions or causal relationships in the fatty acid metabolome. A directed causal network was constructed that revealed 29 significant edges among the 15 nodes (*p* < 0.001). We report that two fatty acid metabolites, palmitoleate and margarate, which originate from dietary intake, together influence every other fatty acid in the network. On the other hand, despite its high connectivity, dihomo-linoleate did not appear to play an important role over the whole fatty acid network. These findings collectively suggest possible strategic entry points for new treatments or preventive modalities against diseases affected by fatty acid metabolites such as cardiovascular disease, diabetes, and obesity. Further studies examining the embedded substructure of the fatty acid metabolite networks in independent population samples would be timely and warranted as we move toward novel postgenomic diagnostics and therapeutics.

## Introduction

Fatty acids are organic molecules that are an integral component of lipid metabolism and dietary fat intake and serve as crucial sources of energy (Castell et al., 1972). In certain cases, blood fatty acid levels can be predictive of future pathology (Markel et al., 2014) or are etiologically related to the onset of common complex diseases such as hepatic steatosis (Hooper et al., 2011). As a result, fatty acids can serve as molecular targets of intervention for prevention as well as treatment of diseases, including the dyslipidemias associated with diabetes (Chehade et al., 2013).

Circulating fatty acids originate from the diet, metabolism of other fatty acids, or are synthesized *de novo* from acetyl-CoA and malonyl-CoA (Nakamura and Nara, 2003). Circulating fatty acid levels are intercorrelated, but the origins of these correlations are complex and not well understood. Gaining a better understanding of the underlying relationships among fatty acid concentrations may help understand the origins, prevention strategies, and treatments of disease.

Consider, for example, two fatty acids, *A* and *B*, and let *B – A* indicate the association or correlation between A and B. A better understanding of the relationship between *A* and *B* means that we want to know whether *A* regulates *B* ($A \rightarrow B$), *B* regulates *A* ($B \rightarrow A$), or some unknown factor regulates both *A* and *B*, thus generating a correlation between *A* and *B* ($A \leftarrow C \rightarrow B$). To distinguish among these three scenarios, an intervention on *A* and/or *B* and subsequent analysis of the response of the other metabolites is usually considered necessary. Genetic mutations that influence *A* or *B* would also allow us to determine the direction of effect, which has been termed Mendelian randomization to connote a naturally randomized intervention (Gray and Wheatley, 1990; Swerdlow et al., 2015). However, to date, the applications of Mendelian randomization have usually considered only one or a few genes and one or a few variables whose directionality is in question (Polfus et al., 2015; Yazdani et al., 2016b).

Recently, Yazdani et al. (2016c) have introduced and applied (Yazdani et al., 2016d) an algorithm, Genome-Directed Acyclic Graphs (GDAG), for determining directions or causal relationships among a large number of variables. The algorithm leverages the principal of Mendelian randomization as applied across the entire genome to identify causal relationships among variables of interest.

To the statistician, Mendelian randomization is equivalent to instrumental variable analysis, and having genome-wide data facilitates creating strong instrumental variables (Yazdani et al., 2016c). To the biomedical scientist, Mendelian randomization facilitates determination of hypothesized causation from mere association.

The aim of the present study was identification and analysis of a statistical causal network among serum fatty acid levels using genome-wide single-nucleotide polymorphism (SNP) data and the GDAG algorithm. Identification of a fatty acid metabolomic-directed network is thought to shed light on points within the network leading to disease prediction and/or intervention. Fifteen mostly long-chain fatty acids were measured as part of an untargeted metabolomic study of serum collected in the fasting state among 2479 African-American (AA) individuals from the Atherosclerosis Risk in Communities (ARIC) study. The results show that two fatty acid metabolites, palmitoleate and margarate, importantly influence every other fatty acid in the network.

## Methods

### Study sample

The results presented here were derived from 2479 AA individuals from the large ARIC cohort study. The ARIC study design was described in detail previously and has a clinical trial registration number of NCT00005131 (The ARIC Investigators, 1989). In brief, a total of 15,792 individuals, predominantly European-Americans and AAs, participated in a baseline visit in 1987–1989, with three additional triennial follow-up visits and a fifth visit in 2011–2013. Because of a dearth of metabolomic data available in AAs and limited funds, we randomly sampled AAs for a population-based study of the serum metabolome at the baseline examination. Focusing on AA individuals from Jackson (MS, USA) in the ARIC cohort allowed us to overcome a host of confounders such as population-to-population and regional dietary variations in the metabolome.

### Metabolomic and SNP measurements

The primary objective of this study was to investigate the causal relationship among 15 serum fatty acid levels measured by untargeted metabolomics (Vinayavekhin and Saghatelian, 2010) from serum samples collected in the fasting state. Fatty acid levels were measured by Metabolon, Inc., (Durham, NC, USA) using a combination of liquid and gas chromatography, followed by mass spectroscopy. Serum samples were extracted and prepared using Metabolon's standard solvent extraction method. The extracted samples were split into equal parts for analysis on complementary GC/MS (gas chromatography mass spectrometry) and LC/MS (liquid chromatography mass spectrometry) platforms. Named compounds were identified by comparison with an in-house-generated authentic standard library that includes retention time, molecular weight, preferred adducts, in-source fragments, and associated fragmentation spectra of the intact parent ion. Fatty acid metabolites were transformed to be normally distributed. Common SNP genotypes at 691,940 variable loci across the genome were measured using the Affymetrix SNP chip array (version 6) using standard instrumentation and methods.

### The GDAG algorithm

Biological granularities are levels of data with different essences, where the direction of effect among them is known. For example, genotype differences in a lower granularity influence interindividual phenotypic variation in an upper level of granularity and not vice versa. Therefore, in a genotype–phenotype network, arrows, which represent direction of effects, go from genotype to phenotype and not the other way around. This kind of data integration framework is established in the concept of instrumental variables, which is called Mendelian randomization in a biomedical setting. The GDAG algorithm (Yazdani et al., 2016c) creates strong instrumental variables from the genome granularity to generate a robust causal network over the observed traits.

The steps of the GDAG algorithm are as follows: First, we note that some SNPs were nearly perfectly correlated (>0.80) with other nearby SNPs so that one SNP can serve as a proxy for many others in the analysis. Therefore, we used the estimated linkage disequilibrium to select a subset of informative SNPs following the algorithm outlined in Yazdani and Dunson (2015).

Second, we obtain strong instrumental variables across the genome using the method of principal components (PCs). The PC analysis applied here had the purpose to extract variations across SNPs and is different from the one applied for population stratification.

Third, we applied the GDAG algorithm using the PCs and the 15 fatty acid metabolites. Eighteen PCs remained in the model at a significant level, 0.001. Using the principal of Mendelian randomization and the PCs from across the genome, we next determine the directionality of the relationships among the fatty acid metabolites.

## Results

**FIG. 1.**
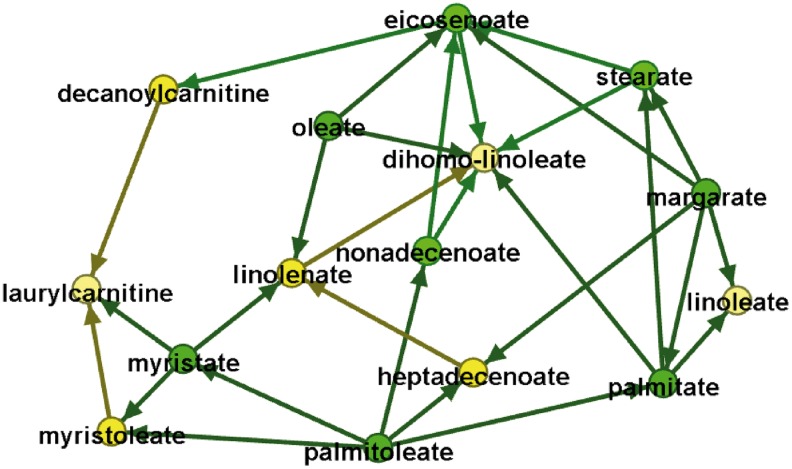
Fatty acid metabolomic causal network. The *nodes* represent observed fatty acid levels and the *arrows* represent causal relationships between each of the two fatty acids.

Supplementary Figure S1 depicts the GDAG over the 15 fatty acid metabolites, including the PCs that remained in the model, where yellow nodes depict the PCs and greens nodes depict the fatty acid metabolites. The number of significant PCs across the 15 traits is 18. There are some metabolites having no PCs, which significantly influence them, and there are some metabolites that are influenced by multiple PCs. The objective of this study is to identify a causal network structure over the set of serum fatty acid metabolites. The genome information in the deeper granularity (i.e., the yellow nodes in Supplementary Fig. S1) is a tool to aid in identifying directionality among the fatty acid metabolites in the upper granularity. Therefore, we delete the genome-wide PCs from the network in Supplementary Figure S1 to better highlight causal relationships among the metabolites; the result is depicted in Figure 1. The number of edges shown in the network in Figure 1 is 29 and the number of nodes is 15, the number of fatty acids under consideration. An arrow between two nodes shows the direction of the flow of information.

**FIG. 2.**
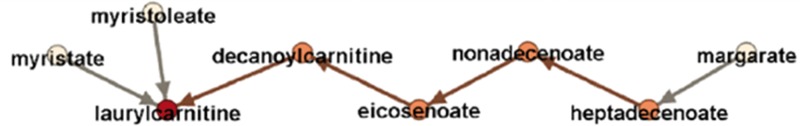
Maximum effect blocking step of the metabolite margarate.

Selected network parameters are represented in Table 1 and show the influence and role of individual nodes in the network. Let the in-degree of a node represent the number of arrows that come into the node and the out-degree of a node represent the number of arrows that go out of the node. A high out-degree reflects substantial influence of that fatty acid on the other fatty acids in the network. A fatty acid with marked in-degree, but zero out-degree, is a point where effects of other fatty acids are blocked. We define the effect blocking step as the number of nodes influenced by one node in one path before the effect is blocked. The maximum number of steps across paths leading out from a node is called the maximum effect blocking step. Figure 2 shows that the effect of the node margarate is blocked at the node laurylcarnitine, yielding a maximum effect blocking step of margarate equal to 5.

**Table 1.** Fatty Acid Metabolomic Network Parameters

*Metabolite*	*Out-degree*	*In-degree*	*Connectivity*	*Max effect blocking steps*
Myristate	3	1	4	2
Myristoleate	1	2	3	1
Palmitate	3	2	5	3
**Palmitoleate**	**5**	**0**	**5**	**5**
**Margarate**	**5**	**0**	**5**	**5**
Heptadecanoate	1	2	3	2
Stearate	2	2	4	3
Oleate	3	0	3	2
Nonadecanoate	2	1	3	3
Eicosenoate	2	4	6	1
Linoleate	0	2	2	0
Linolenate	1	3	4	1
Dihomo-linoleate	0	6	6	0
Decanoylcarnitine	1	1	2	1
Laurylcarnitine	0	3	3	0

Some of the network parameters are tabulated above. The bold highlighted metabolites, palmitoleate and margarate, have the maximum out-degree, zero in-degree, and the highest maximum effect blocking steps in the causal network, which represent the metabolites with important roles over the network.

The topology and directionality of the GDAG allow one to generate hypotheses about where an intervention on one fatty acid would likely propagate to other fatty acids within the network. Fatty acids with a low number of effect blocking steps and low level of out-degree connectivity are not predicted to influence other fatty acids across the network. For example, eicosenoate and dihomo-linoleate are not predicted to play important roles in the fatty acid network, despite their high connectivity (Fig. 1). These two fatty acids are greatly influenced or regulated by others in the network, but they have little influence on the other fatty acids. Dihomo-linoleate, in particular, has zero out-degree, meaning that it does not influence any other fatty acid in the network. In sharp contrast, palmitoleate and margarate together regulate every other fatty acid in the network directly or indirectly and are not influenced by other fatty acids in the network (Fig. 1). The maximum effect blocking step for these two metabolites is 5, the largest in the network. However, the effect of margarate is blocked before reaching myristate, and thus an intervention on margarate is expected to not have an effect on the level of myristate.

**FIG. 3.**
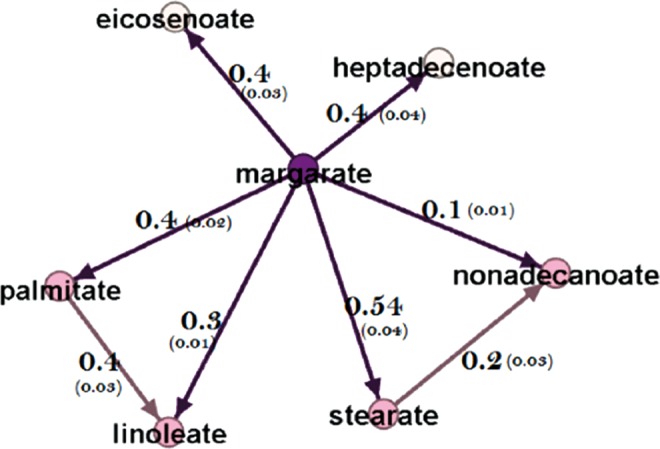
Estimated effect sizes (and standard errors). The effect of metabolite margarate on the local metabolites in the fatty acid network.

One of the most important features of a directed network is the ability to estimate causal effect sizes in an observational study. The generated network represents the causal relationships among fatty acids. Therefore, confounders can be distinguished from the set of covariates (Yazdani and Boerwinkle, 2015) to estimate the causal effect of one metabolite on another (Supplementary Figure S2). In this study, for example, the effects of margarate on the local metabolites are measured and represented in Figure 3. The interested readers are referred to the Supplementary Data and (Yazdani et al., 2016a) for details about effect size estimation.

## Discussion

Fatty acids are an important and heterogeneous source of energy within all cell types and are synthesized within the body and ingested as part of a regular diet. The average American consumes about 26% of calories as fatty acids. The American Heart Association recommends a diet rich in monounsaturated fatty acids relative to polyunsaturated fatty acids and the elimination of trans fatty acids (Dolecek and Granditis, 1991; Kris-Etherton et al., 2002). We selected a set of fatty acid metabolites and identified causal relationships among them using the GDAG algorithm (Yazdani et al., 2016c). A statistical causal network provides not only the relationships among the metabolites but also directions of those relationships. Having the fatty acid causal network identified, we can have optimal inference by systematically moving through the network to see the consequences of an intervention at a specific node on the rest of the network. We then compare maximum effect blocking steps as well as out- and in-degree properties of the metabolites. Despite high connectivity, the metabolites, eicosenoate and dihomo-linoleate, are not predicted to be influential metabolites in the fatty acid network due to low out-degree and low effect blocking steps. In contrast, the metabolites, palmitoleate and margarate, together regulate every other metabolite in the network directly or indirectly. They play important roles among the fatty acid metabolites due to high out-degree, zero in-degree, and high maximum effect blocking steps.

Based on available biological knowledge, the metabolites, palmitoleate and margarate, originate directly from dietary intake and palmitoleate is also synthesized *de novo*, and it may be that the health effects of these fatty acids depend on their origin (Stempfle et al., 2016). Palmitoleate is unusual for a fatty acid in that it appears to be highly compartmentalized within tissues (Hodson and Karpe, 2013). Based on these results, we may hypothesize that dietary intervention on these two metabolites would be predicted to have an influence across the fatty acid network.

In the GDAG algorithm, the necessary base knowledge is the causal relationship between the two levels of granularity, that is, the inherited genome variation is a causal factor of the fatty acid metabolome. Therefore, the GDAG algorithm is particularly informative for novel phenotypes, such as the metabolome, where there is not extensive information about the detailed relationships among them and there is not sufficient information about the relationships between individual genome variants and particular metabolites. Since strong instrumental variables are applied from the genome granularity, detailed knowledge about the relationship between one gene and a metabolite is not necessary for the GDAG algorithm and the algorithm is not biased by partial genome information (Yazdani et al., 2016c).

Rapid advances in network biology indicate that biological processes are influenced by a common set of rules and offer a new conceptual framework that may potentially alter our view of biology and disease pathology. Understanding the topology and dynamics of the relationships among individual omics measures is a key challenge for science in the 21st century. In this study, we tried to meet this challenge using causal networks. Optimal inference decisions can be made based on the causal network theory and causal relationships between different entities as a graph model (Kim et al., 2010).

## Conclusions and Expert Outlook

Using information from across the genome and the GDAG algorithm enabled us to identify robust and novel causal relationships among fatty acid metabolites in the present study. Analyzing these causal relationships provides insight into potential points of intervention or prediction. The metabolites, palmitoleate and margarate, are identified to have important roles across the whole fatty acid network. In contrast, eicosenoate and dihomo-linoleate are not predicted to be influential metabolites in the network, but they may be good predictors.

These findings collectively suggest possible strategic entry points for future interventions aimed at the development of new treatments or preventive modalities against disease affected by fatty acid metabolites, such as cardiovascular disease, certain forms of cancers, diabetes, and obesity.

## Supplementary Material

Supplemental data

## Abbreviations Used

AA: African-American
ARIC: Atherosclerosis Risk in Communities
GDAG: Genome-Directed Acyclic Graph
PC: principal component
SNP: single-nucleotide polymorphism
