# Supplementary material for: A Causal Network Analysis of the Fatty Acid Metabolome in African-Americans Reveals a Critical Role for Palmitoleate and Margarate
Source: OMICS. 2016 Aug 1;20(8):480–4. doi: 10.1089/omi.2016.0071 (PMC4982951; doi:10.1089/omi.2016.0071)
Supplement: Supplemental data [file Supp_Data.pdf]

Supplementary Data

Steps to Apply the Genome-Directed Acyclic Graph Algorithm

First, we note that some nearby single-nucleotide polymorphisms (SNPs) were nearly perfectly correlated (>0.80) with others so that one SNP can serve as a proxy for many others in the analysis. Therefore, we used the estimated linkage disequilibrium to select a subset of informative SNPs following the algorithm outlined in Yazdani and Dunson (2015). Second, we obtain the principal components (PCs) separately over each chromosome since the chromosomes are independent. The first few PCs that explain over 90% of the variation in each chromosome were selected. Over all of the chromosomes, 286 PCs were selected for the metabolomic network analyses. Third, we applied the Genome-Directed Acyclic Graph (GDAG) algorithm over the 286 genome-wide PCs and the 15 metabolites. The GDAG algorithm first uses partial correlations to generate the topology of the network over the 15 fatty acid phenotypes and the genome-wide PCs, 18 of which remained in the model at significant level 0.001. Using the principal of Mendelian randomization and strong instrumental variables across the genome, we next determine the directionality of the relationships among the fatty acid metabolites.

The Causal Parameter

To have causal inference in observational settings, we need to clarify relationships among variables and influences underlying the study observations, which is called the illumination of the assignment mechanism and is a causal assumption (Yazdani and Boerwinkle, 2014; Yazdani et al., 2016). The causal assumption/causal parameter is formalized as $AM(K_R)$, which stands for illumination of assignment mechanism (AM) given any knowledge about response (K_R); here response variables are fatty acids. In this article, the AM is formalized by a causal network. Attempts to obey this causal assumption connote the parallel nature with an intervention. By illumination of the assignment mechanism/causal network, we can identify confounders to measure causal effects as if we have an intervention. This assumption is formally and explicitly represented through the causal parameter $AM(K_R)$. Equations to estimate the causal effects are conditioned on the causal parameter, $AM(K_R)$, to represent the fact that the estimates are given the generated causal network and formally represent the causal assumption.

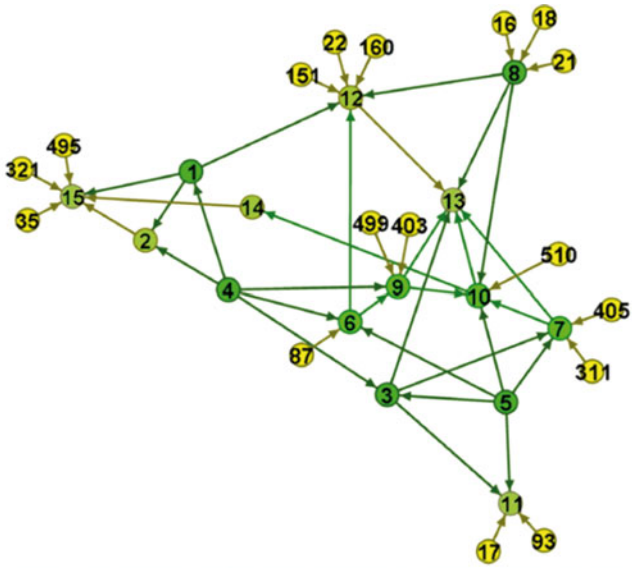

SUPPLEMENTARY FIG. S1. Fatty acid metabolomic GDAG. *Green nodes* correspond to fatty acid metabolites and *yellows* correspond to genome-wide PCs. GDAG, Genome-Directed Acyclic Graph; PCs, principal components.

Causal Effect Estimation

Given the fatty acid network in Figure 1, we identify confounders to measure causal effects. Assume F_i stands for the ith fatty acid, and assume we are interested in the effect of F_1 on F_2. From the generated network in Figure 1, F_4 influences both F_1 and F_2, that is, two arrows come to nodes F_1 and F_2 from the same node F_4. Therefore, variable F_4 is a confounder to measure the effect of F_1 on F_2. This is a good example to see how variables are involved in the mechanism of assigning different levels to a specific variable.

To measure the effect of F_1 on F_2, we carry out the following steps:

1. Remove the effect of variations in F_4 on F_1 by the following equation:

$$F_1|AM(K_R) = \theta_{41}F_4 + U$$

where U stands for variations in F_1 independent of F_4. For this computation, the related part of the network in Figure 1 is illustrated in Supplementary Figure S2. For better illustration, variable U is also depicted in the network in Supplementary Figure S2.

2. Fit F_2 on U:

$$F_2|AM(K_R) = \theta_{12}U + U'$$

where the coefficient θ_{12} has causal interpretation since we assume the independence $U \perp U'$ is held and is assumed the causal effect of an intervention on F_1 that is expected to influence F_2.

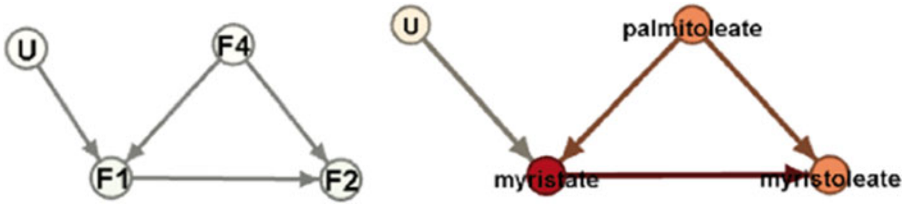

SUPPLEMENTARY FIG. S2. A subset of the fatty acid metabolomic network in Figure 1 to measure the effect size, myristate,F_1, on myristoleate, F_2. The metabolite palmitoleate, F_4, is a confounder. U includes all factors with influence on F_1 independent from F_4. The two networks are the same; the name of nodes in the *left* is replaced with the corresponding metabolite in the *right*.
